# Microstructural Infarct Border Zone Remodeling in the Post-infarct Swine Heart Measured by Diffusion Tensor MRI

**DOI:** 10.3389/fphys.2018.00826

**Published:** 2018-08-22

**Authors:** Geoffrey L. Kung, Marmar Vaseghi, Jin K. Gahm, Jane Shevtsov, Alan Garfinkel, Kalyanam Shivkumar, Daniel B. Ennis

**Affiliations:** ^1^Department of Radiological Sciences, David Geffen School of Medicine, University of California, Los Angeles, Los Angeles, CA, United States; ^2^Department of Bioengineering, University of California, Los Angeles, Los Angeles, CA, United States; ^3^Cardiac Arrhythmia Center, David Geffen School of Medicine, University of California, Los Angeles, Los Angeles, CA, United States; ^4^Department of Medicine (Cardiology), David Geffen School of Medicine, University of California, Los Angeles, Los Angeles, CA, United States; ^5^Department of Computer Science, University of California, Los Angeles, Los Angeles, CA, United States; ^6^Biomedical Physics Interdepartmental Program, David Geffen School of Medicine, University of California, Los Angeles, Los Angeles, CA, United States

**Keywords:** cardiac computational models, diffusion tensor MRI, border zone, cardiac remodeling, cardiac electromechanics

## Abstract

**Introduction:** Computational models of the heart increasingly require detailed microstructural information to capture the impact of tissue remodeling on cardiac electromechanics in, for example, hearts with myocardial infarctions. Myocardial infarctions are surrounded by the infarct border zone (BZ), which is a site of electromechanical property transition. Magnetic resonance imaging (MRI) is an emerging method for characterizing microstructural remodeling and focal myocardial infarcts and the BZ can be identified with late gadolinium enhanced (LGE) MRI. Microstructural remodeling within the BZ, however, remains poorly characterized by MRI due, in part, to the fact that LGE and DT-MRI are not always available for the same heart. Diffusion tensor MRI (DT-MRI) can evaluate microstructural remodeling by quantifying the DT apparent diffusion coefficient (ADC, increased with decreased cellularity), fractional anisotropy (FA, decreased with increased fibrosis), and tissue mode (decreased with increased fiber disarray). The purpose of this work was to use LGE MRI in post-infarct porcine hearts (*N* = 7) to segment remote, BZ, and infarcted myocardium, thereby providing a basis to quantify microstructural remodeling in the BZ and infarcted regions using co-registered DT-MRI.

**Methods:** Chronic porcine infarcts were created by balloon occlusion of the LCx. 6–8 weeks post-infarction, MRI contrast was administered, and the heart was potassium arrested, excised, and imaged with LGE MRI (0.33 × 0.33 × 0.33 mm) and co-registered DT-MRI (1 × 1 × 3 mm). Myocardium was segmented as remote, BZ, or infarct by LGE signal intensity thresholds. DT invariants were used to evaluate microstructural remodeling by quantifying ADC, FA, and tissue mode.

**Results:** The BZ significantly remodeled compared to both infarct and remote myocardium. BZ demonstrated a significant decrease in cellularity (increased ADC), significant decrease in tissue organization (decreased FA), and a significant increase in fiber disarray (decreased tissue mode) relative to remote myocardium (all *p* < 0.05). Microstructural remodeling in the infarct was similar, but significantly larger in magnitude (all *p* < 0.05).

**Conclusion:** DT-MRI can identify regions of significant microstructural remodeling in the BZ that are distinct from both remote and infarcted myocardium.

## Introduction

Computational modeling of cardiac electromechanics (Krishnamoorthi et al., 2014) can provide mechanistic insight to normal and abnormal cardiac function and electrical wave propagation (Ponnaluri et al., 2016). Chronic myocardial infarction remains a substantial risk factor for both mechanical heart failure and fatal electric rhythm abnormalities. The post-infarct heart is characterized by three distinct regions, including the remote (“normal”) myocardium, the dense infarcted scar, and the border zone (BZ) transition region between remote and infarcted myocardium. The infarct BZ is known as a site for electromechanical property transition.

Myocardial fibrosis as a consequence of post-infarct remodeling increases apparent tissue stiffness and decreases anisotropy. Increases in tissue stiffness are implicated in both abnormal diastolic filling and abnormal systolic contraction, ultimately fomenting heart failure. Myocardial fibrosis also disrupts normal electrical wave front propagation, which contributes to the initiation of fatal ventricular arrhythmias (de Bakker et al., 1988, 1993; Morita et al., 2009). In particular, the infarct border zone (BZ) facilitates slow conduction, reentry phenomena, and is implicated in arrhythmogenesis (Anversa et al., 1985; Ursell et al., 1985; de Bakker et al., 1988; Miragoli et al., 2007). Furthermore, anisotropic tissue conduction at epicardial border zones has been shown to influence the occurrence of reentry (Dillon et al., 1988). Methods to identify the BZ for incorporation into computational models of cardiac electromechanics, however, are not currently well established.

Subsequent to administration of a gadolinium-based contrast agent, T1-weighted late gadolinium enhanced (LGE) magnetic resonance imaging (MRI) is recognized as the gold standard for non-invasive myocardial infarct mapping (Kim et al., 2000; Karamitsos et al., 2009; Schelbert et al., 2010). In LGE MRI the slow contrast washout time from the extracellular space gives rise to hyper-enhanced signal intensity (SI) within the infarct (Kim et al., 1996). The adjacent BZ is characterized by a mixture of replacement fibrosis and viable myocytes within the tissue and, as a consequence of partial volume effects, yields an intermediate SI in LGE MRI (Anversa et al., 1985; Schelbert et al., 2010). LGE MRI, however, only indirectly indicates the presence of microstructural remodeling, especially in the infarct and BZ as it only directly reports the presence of the contrast agent. The extent of microstructural remodeling within the LGE identified BZ has not been previously been characterized.

Diffusion tensor MRI (DT-MRI) quantifies the self-diffusion tensor of water undergoing Brownian diffusion within each imaging voxel. This enables the direct quantitative evaluation of microstructural remodeling (e.g., direct and quantitative changes to the tissue microenvironment). Microstructural remodeling is frequently reported using tensor invariants, which saliently characterize important shape attributes of microstructural diffusion and are established as a tool for quantifying differences in regional microstructure (Ennis and Kindlmann, 2006). Complementary information is found in the eigenvectors, which accord with the predominant cardiomyocyte orientation and myolaminar sheetlet orientations (Kung et al., 2011).

A particularly useful set of microstructural remodeling metrics (tensor invariants) consists of the DT's: (1) *apparent diffusion coefficient* (ADC, [mm^2^/s]), which measures the overall magnitude of isotropic diffusion and increases with decreasing tissue cellularity (Ellingson et al., 2010); (2) *fractional anisotropy* (FA, unitless on [0, 1]), which quantifies the magnitude of anisotropic diffusion and decreases with increasing fibrosis(Wu et al., 2007); and (3) *tissue mode*(Ennis and Kindlmann, 2006) (unitless on [−1, 1]), which gauges the kind of tissue anisotropy with mode values near zero indicating orthotropic diffusion indicative of sheet-like structures; mode values near +1 indicating rod-like tissue organization; and mode values near −1 indicating planar or pancake-like tissue organization.

The objective of this study was to quantify and compare microstructural remodeling in the remote, BZ, and infarcted myocardium of the post-infarct swine using DT-MRI. We hypothesized that microstructural remodeling (changes in ADC, FA, and tissue mode) within the BZ and infarct will constitute a significantly different microstructural environment compared to remote myocardium.

## Materials and methods

### Porcine heart preparation

Animal handling and care followed the recommendations of the National Institutes of Health Guide for the Care and Use of Laboratory Animals and the University of California, Los Angeles Institutional Animal Care and Use Committee. Animal protocols were approved by the University of California, Los Angeles Chancellor's Animal Research Committee.

Following a 12-h fasting period, the swine for this study were intramuscularly injected with 1.4 mg/kg Telazol, and then intubated. General anesthesia was maintained with inhaled 2.5% isoflurane. Seven adult female Yorkshire pigs (40–55 kg) (*N* = 7) underwent closed chest myocardial infarction via balloon occlusion and subsequent reperfusion of the left circumflex artery (LCx). An obtuse marginal branch of the LCx was occluded for 150 min with an angioplasty balloon via the retrograde aortic approach using a sheath from the right femoral artery. Evolving infarction was confirmed via ST segment elevation as assessed by continuous electrocardiogram monitoring.

After 6 to 8 weeks, the animals were intubated and placed under general anesthesia as above. Gd-DTPA was injected (0.1 mmol/kg) and allowed to circulate for 15 min before euthanizing with a lethal dose of KCl. Normal adult female Yorkshire pigs (35–50 kg) (*N* = 7) served as the control group undergoing an identical euthanasia procedure without infarct induction.

After sacrifice, each heart was excised by cutting the great cardiac vessels, rinsed with saline and suspended by the root of the aorta in a saline filled container. With the heart suspended, a high viscosity silicone rubber injection compound (Ready-Press Polyvinylsiloxane, Microsonic Inc., Ambridge, PA) was injected first through the pulmonary vein to fill the left ventricle and left atrium then through the superior vena cava to fill the right ventricle and right atrium, in order to maintain an approximate end diastolic cardiac anatomy (Kung et al., 2011). The heart was then removed from saline and placed in a one-liter plastic cylindrical container filled with a magnetic susceptibility matched fluid (Fomblin Y-LVAC 6-06, Solvay Solexis, West Deptford, NJ). The heart was held in place within the container using open-cell foam and oriented to grossly align with the long axis of the container and subsequently the MRI scanner. The combination of the silicone rubber injection compound, magnetic susceptibility matched fluid, and open-cell foam maintains hold the heart rigidly in place during long scan times. These materials also produce very low MRI signals, which significantly facilitates image segmentation.

### *Ex vivo* magnetic resonance imaging

Imaging was performed using a 3 Tesla (Trio, Siemens AG, Munich, Germany) scanner and a 12-channel head coil. A 3D gradient echo LGE MRI sequence was used with the following pulse sequence parameters: TR/TE = 4.24/9.35 ms, flip angle = 18.5°, bandwidth = 260 Hz/pixel, 9 averages, and scan time = 2:18 (HH:MM). The in-plane imaging resolution was 0.33 mm × 0.33 mm × 0.33 mm (~550 myocytes per voxel) obtained by using a 384 × 384 × 256 encoding matrix and a 128 × 128 × 85.33 mm imaging volume. All MRI exams began within 2 h of sacrifice to ensure Gd-DTPA contrast did not diffuse significantly away from the infarct (Schelbert et al., 2010).

Immediately after LGE imaging, spatially co-registered DT-MRI was performed. A two-dimensional, diffusion weighted, readout-segmented echo-planar pulse sequence (Porter and Heidemann, 2009) was used to acquire DT-MRI data. The following pulse sequence parameters were used for all experiments: TE/TR = 76 ms/6,800 ms, *b*-value = 1,000 s/mm^2^, 30 non-collinear diffusion gradient encoding directions, one non-diffusion weighted null direction, 15 readout segments, bandwidth = 439 Hz/pixel, and 8–10 averages. The in-plane imaging resolution was 1 mm × 1 mm × 3 mm (~42,000 myocytes per voxel) obtained by using a 150 × 150 encoding matrix, 43–44 slices and a 150 × 150 × 129–132 mm imaging volume. The total imaging time for each diffusion weighted volume was 3.4 min, for a total DT-MRI acquisition time of 7:00–8:50 (HH:MM) per heart.

Diffusion tensors were reconstructed from the diffusion weighted images using linear regression and custom Matlab (The Mathworks, Natick, MA) code. ADC, FA, and mode (Ennis and Kindlmann, 2006) were calculated for each imaging voxel's diffusion tensor. The diffusion tensors were visualized directly with superquadric glyphs, which are 3D surfaces that depict the tensor's shape and orientation and highlight regional organization and remodeling (Ennis et al., 2005).

### LGE segmentation and registration to DT-MRI

#### Segmentation

**Figure 1:**
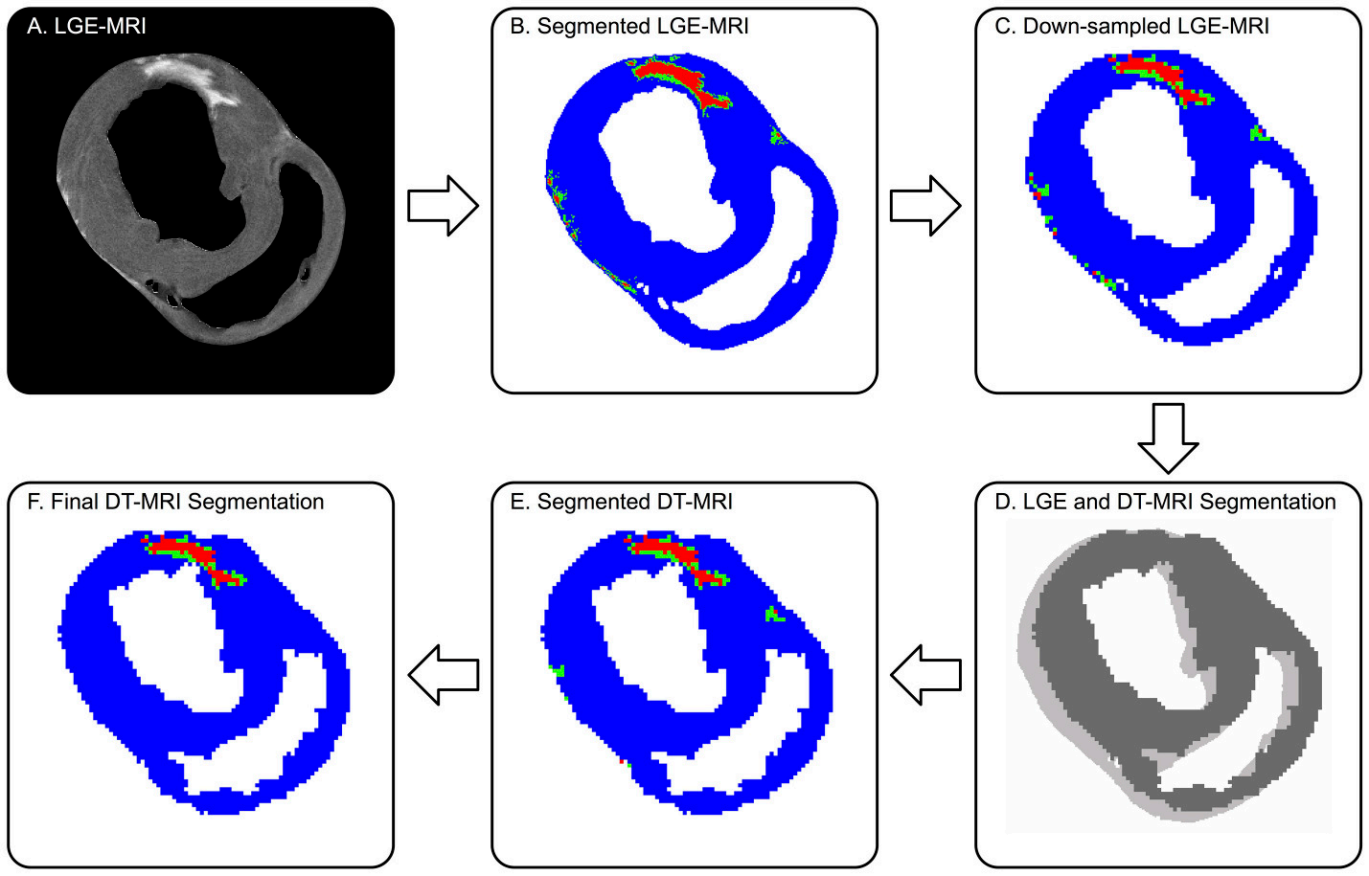
Defining remote (blue), borderzone (green), and infarcted (red) myocardium on DT-MRI from co-registered LGE MRI. **(A)** Original high-resolution LGE MRI shows a distinctly bright focal infarct. **(B)** The threshold segmented LGE image is **(C)** down-sampled to match the DT-MRI resolution, then **(D)** co-registered to DT-MRI via 2D cross-correlation. **(E)** The registered LGE segmentation mask is superimposed onto the corresponding myocardial mask of the DT-MRI data. **(F)** Final DT-MRI segmentation refined by excluding islands of high signal intensity consisting of three or less voxels (9 mm^3^) and removing BZ signal intensities greater than three voxels (3 mm) away from infarct. Note, the LGE to DT-MRI registration required a single pixel-level shift.

LGE images (Figure 1A) were segmented into remote, BZ, and infarcted myocardium and registered to the diffusion tensor images to enable analysis of microstructural remodeling using the validated procedure of Schelbert et al. (2010). First, LGE images were averaged in the slice direction to match the slice thickness of corresponding DT-MRI data. Myocardial voxels were designated as remote, BZ, or infarct based on LGE image signal intensity (SI) thresholds defined for each heart (Ashikaga et al., 2007; Schelbert et al., 2010; Tao et al., 2010). Regions of interest in remote myocardium (myocardial voxels with low SI) and infarct (myocardial voxels with high SI) were drawn in each heart to calculate the SI mean and standard deviation (SD) in each region for each infarcted heart. In accordance with the method used by Schelbert et al. (2010), segmentation of infarct and remote myocardium was defined starting with a threshold halfway between the mean SI of remote myocardium and the mean SI of infarcted myocardium on a per heart basis. The BZ was defined as voxels with SI below the halfway SI level, but greater than two SDs above mean remote myocardium (Figure 1B).

#### Registration

LGE and DT-MRI studies were performed back-to-back without adjusting the position of the tissue, but small spatial shifts still occurred over the long scan times. Therefore, 3D rigid-body registration was employed between a binary mask created using SI thresholds of the myocardium in the LGE images and binary a mask of the myocardium from the DT-MRI data created using a tensor-based segmentation method (Gahm et al., 2013). The binary LGE masks were down-sampled in the in-plane directions using bicubic interpolation to match the resolution of DT-MRI (Figure 1C). LGE and DT-MRI data were registered first in the through-plane direction by aligning the LV apex of both binary mask image sets, then registered via rigid translation in the in-plane direction using two dimensional cross-correlations in Matlab (Figure 1D). The registered LGE mask was then applied to the DT-MRI data to label each voxel as remote, BZ or, infarct (Figure 1E). The DT-MRI segmentation was further refined by excluding infarct labeled voxels when the connected regions of infarct consisted of three or less voxels (Figure 1F), similar to Tao et al. (2010). BZ segmentations were also refined using morphologic operations to exclude voxels designated as BZ that were greater than three voxels away from infarct labeled regions.

### Statistical analysis

The central hypothesis of this work is that microstructural remodeling (changes in ADC, FA and mode) within the BZ and infarct constitute a significantly different microstructural environment compared to remote myocardium. Testing this hypothesis required developing the statistically appropriate methods for characterizing significant microstructural remodeling because pixel-based imaging data is spatially correlated and the underlying distribution of the measurement data is non-Gaussian distributed. The key statistical analysis steps include: (1) spatial decorrelation of the data by the auto-correlation length; (2) application of distribution-independent bootstrapped analogs to conventional Gaussian statistical methods; (3) application of boot-strapped analogs of the common *t*-test to enable comparison of medians from non-Gaussian distributions; and (4) use of the boot-strapped analog repeated measures ANOVA to compare groups.

First, the use of inferential statistics requires statistically independent samples. The highly-organized arrangement of myocytes within normal myocardium, however, results in high spatial correlation of the myocardial diffusive properties. For example, adjacent pixels have very similar or spatially correlated tissue properties. This leads to statistically non-independent local diffusion tensors and tensor invariants. To produce statistically independent data points—ADC, FA, and mode were spatially de-correlated in three dimensions within remote, BZ, and infarct regions via decimation by each region's auto-correlation length (Gahm et al., 2012).

Second, the standard formula-based statistical tests (e.g., *t*-test or ANOVA) require data to be approximately Gaussian in distribution and to have equal variances between comparison groups. The distribution of tensor invariant data, however, is non-Gaussian with unequal variances across different populations (Kung et al., 2011). Therefore, statistical significance tests were performed using bootstrap methods (Gahm et al., 2012).

Third, in order to compare the statistical distributions of tensor invariants between remote, BZ, and infarcted myocardium, we produced bootstrapped histograms by sampling 1,000 times with replacement from the segmented and spatially de-correlated data to define 95% confidence intervals (CIs) within each of 32 histogram bins. When comparing two regions (e.g., remote vs. BZ), if the 95% CIs of the two regions do not overlap within a histogram bin, then the two regions are significantly different within the invariant range of that bin. Similarly, if the 95%-CIs of the bootstrapped medians for two regions within individual hearts do not overlap, then the two regions are significantly different.

Last, to test whether DT invariants significantly remodeled between remote, BZ, and infarct regions for pooled data from all infarcted hearts, we performed a bootstrap analog to repeated measures ANOVA of the de-correlated data (Lazic, 2010). Remote myocardium in infarcted hearts was also compared to myocardium in normal control hearts using a two-group comparison of the medians of de-correlated data. Bootstrapped repeated measures ANOVA and two-group comparisons were performed using the R programming language (http://www.r-project.org), where *p* < 0.05 was regarded as statistically significant. When reporting image quality and auto-correlation lengths, results are reported as mean ± SD.

## Results

### Infarct evaluation

**Figure 2:**
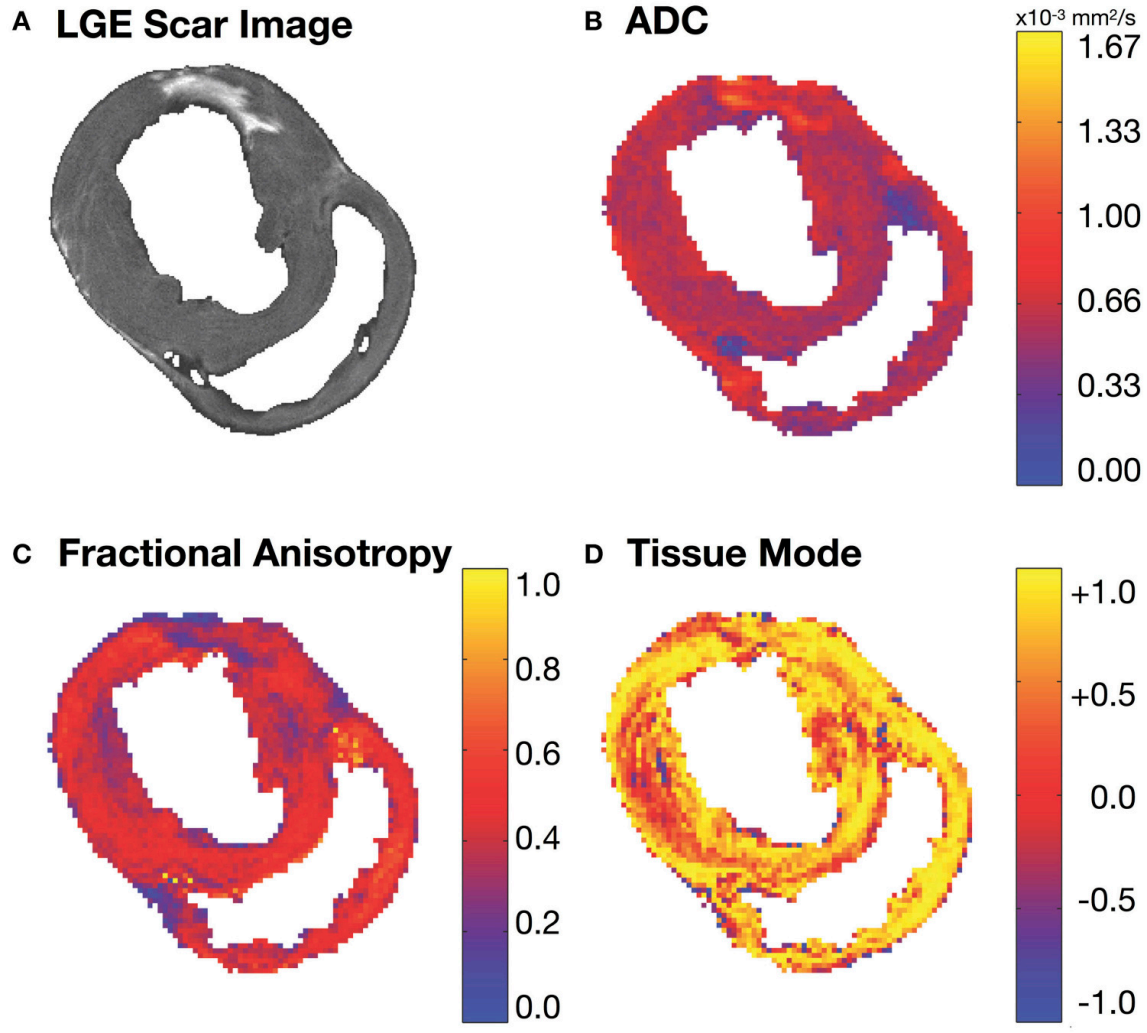
**(A)** Short-axis LGE slice and the corresponding **(B)** apparent diffusion coefficient (ADC), **(C)** fractional anisotropy (FA) and **(D)** tissue mode maps. Regions of increased ADC **(B)** and decreased FA **(C)** match similar regions of hyper-intense SI in the corresponding LGE MRI slice **(A)**.

Balloon occlusion of the LCx resulted in chronic infarcts that exhibited replacement fibrosis as evidenced by the elevated SI in the LGE images (Figures 1A, 2A). Infarcts regions were predominantly located in the inferior and/or inferoseptal basal to apical LV wall.

### Image quality and registration

The mean signal-to-noise ratios (SNR) for the LGE MRI experiments were calculated from each heart by selecting a region of interest (Mewton et al., 2011) in remote/normal myocardium and dividing it by the SD of an ROI of equal area in the background of the same slice for five equally spaced slices within each heart. The mean SNRs from the DT-MRI experiments were calculated from the non-diffusion weighted images for each heart in the same manner as the LGE images. The signal-to-noise ratio of the high resolution LGE images for all hearts was 10 ± 2. The SNR of the non-diffusion weighted images of the DT-MRI was 59 ± 15. 3D rigid-body registration of LGE and DT-MRI data resulted in shifting of the LGE data by 0.8 ± 0.9 and 1.2 ± 1.5 mm in the x- and y-directions (in-plane) respectively and 0.2 ± 0.5 mm in the z-direction (through-plane), which results in sub pixel-level registration differences.

### Data de-correlation

Auto-correlation lengths were 3.0 ± 0.6 voxels (3.0 ± 0.6 mm) in the in-plane x- and y-directions and 1.8 ± 0.2 voxels (5.4 ± 0.6 mm) in the through-plane z-direction for normal hearts and remote myocardium in infarcted hearts. Auto-correlation lengths in the BZ were 1.2 ± 0.1 voxels (1.2 ± 0.1 mm) in the x- and y-directions and 1.1 ± 0.2 voxels (3.3 ± 0.6 mm) in the z-direction. Auto-correlation lengths in the infarct region were 1.7 ± 0.2 voxels (1.7 ± 0.2 mm) in the x- and y-directions and 1.2 ± 0.1 voxels (3.6 ± 0.3 mm) in the z-direction. The mean values were rounded for data de-correlation.

### Visualization of microstructural remodeling

**Figure 3:**
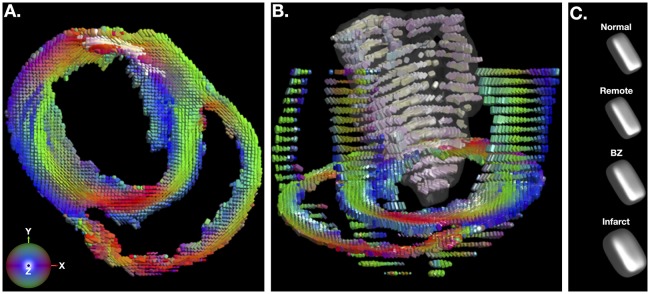
**(A)** Short-axis depiction of diffusion tensor shape and orientation rendered with superquadric glyphs. The long-axis of each glyph is aligned with the primary eigenvector of the diffusion tensor at each voxel. Glyphs are color coded by the primary eigenvector direction with red grossly aligning with the left-right direction, green with the up-down direction, and blue with the through plane direction. The brightness of glyphs in the infarct is enhanced for contrast. **(B)** Short-axis, long-axis and whole-infarct depiction of diffusion tensor shape and orientation rendered with superquadric glyphs. **(C)** Superquadric glyphs of the diffusion tensor shape from normal hearts and remote, borderzone (BZ) and infarcted regions of the heart using the median values of ADC, FA, and tissue mode from Table 1. Supplementary Movie 1 is available in the Supplementary Material.

Figure 2A depicts a representative short-axis LGE slice from an infarcted heart. Corresponding DT invariant maps are shown in Figures 2B–D and show an increase in ADC (Figure 2B), a decrease in FA (Figure 2C), and little apparent change in tissue mode (Figure 2D) within the BZ and infarct regions compared to remote myocardium. Figure 3A depicts, in three dimensions, diffusion tensor remodeling within a short-axis slice, a long-axis slice, and the entire infarct highlighted by a transparent isosurface. Each superquadric glyph's long-axis aligns with each voxel's primary eigenvector and is color coded by mapping the primary eigenvector's components to red-green-blue colormap. The brightness of the infarct glyphs is increased for contrast. Supplementary Movie 1 is available in the Supplementary Material. Figure 3B depicts the same short-axis slice seen in Figure 3A. Figure 3C depicts the diffusion profile in normal, remote, BZ, and infarcted myocardium by rendering the median microstructural tensors using superquadric glyphs. Superquadric glyphs from the infarct are visibly larger (higher ADC) and more isotropic (lower FA) than the glyphs represented by median invariant values from normal and remote myocardium. Glyphs within the BZ of all infarcted hearts show an intermediate size (intermediate ADC) and intermediate isotropy (intermediate FA) compared to the infarct, normal, and remote myocardium glyphs.

### Quantitative evaluation of microstructural remodeling

**Figure 4:**
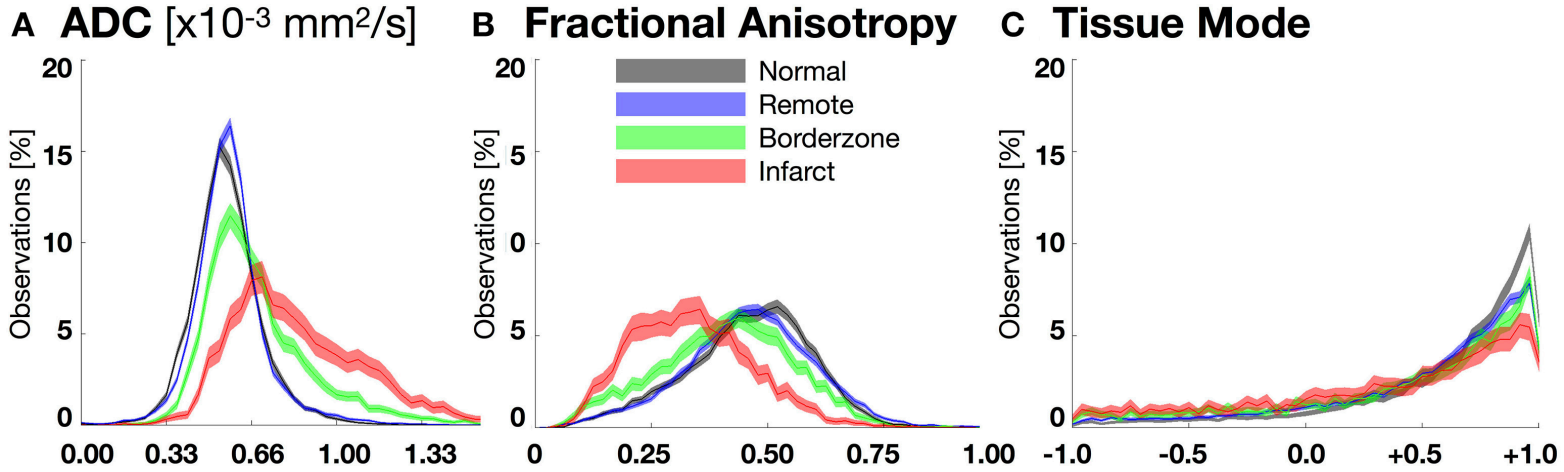
Bootstrapped histograms with 95%-CIs (for each bin) for each segmented region (normal myocardium—black, remote—blue, borderzone—green, and infarct—red) using spatially de-correlated data pooled from all hearts for **(A)** apparent diffusion coefficient (ADC), **(B)** fractional anisotropy (FA), and **(C)** tissue mode. Non-overlapping regions between histograms reveal significant differences within a given bin. Significant microstructural remodeling (increase in the median ADC and decrease in median FA) is evident transitioning from normal to remote to borderzone to infarct histograms. Mode changes suggest an increase in fiber disarray (remodeling toward lower tissue mode values) within the borderzone and infarct regions.

LGE based segmentation of the DT-MRI data revealed significant differences in ADC and FA data between all pairwise comparisons of remote, BZ, and infarct regions within each infarcted heart (Figure 4, Table 1). The BZ within each individual heart is characterized by a significant increase in ADC and significant decreases in both FA and decrease in tissue mode relative to remote myocardium. The infarct region within each heart is characterized by an even larger and significant increase in ADC and significant decreases in both FA and mode.

**Table 1:** Pooled tensor invariant medians and bootstrapped 95%-CIs of the medians for normal, remote, border zone (BZ), and infarcted myocardium.

**Region**	**Median ADC [ × 10^−3^ mm2/s]**	**95%-CI of median ADC [ × 10^−3^ mm2/s]**	**Median fractional anisotropy**	**95%-CI of median FA**	**Median tissue mode**	**95%-CI of median mode**
Normal	0.563	[0.560, 0.565]	0.470	[0.467, 0.473]	0.743	[0.736, 0.749]
Remote	0.573^†^	[0.572, 0.577]	0.464^†^	[0.462, 0.466]	0.666^‡^	[0.660, 0.672]
BZ	0.647^†^	[0.640, 0.650]	0.417^†^	[0.412, 0.421]	0.621	[0.603, 0.635]
Infarct	0.797^†^	[0.787, 0.807]	0.330^†^	[0.325, 0.336]	0.515	[0.495, 0.538]

† *p < 0.0001 for bootstrap analog to repeated measures ANOVA*.

‡ *p = 0.02 for two-group comparison*.

Figure 4 depicts pooled histograms with bootstrapped 95%-CIs of the de-correlated DT invariant data for each segmented region. DT invariant medians and their bootstrapped 95% CIs for each pooled region (normal, remote, BZ, and infarct) are listed in Table 1. Results from the bootstrapped analog to repeated measures ANOVA revealed significant differences across remote, BZ, and infarct regions for all hearts when comparing ADC (*p* < 0.0001) and FA (*p* < 0.0001), but were not significant for mode (*p* = 0.47). In a comparison of normal myocardium from control swine and remote myocardium from infarcted swine, two-group comparisons of the DT invariants did not reveal significant differences between median values of ADC (*p* = 0.18) and FA (*p* = 0.51), but did show a significant decrease in mode (*p* = 0.02) from normal to remote myocardium.

## Discussion

Replacement fibrosis within an infarct significantly alters the electrophysiological and mechanical properties of the myocardium, leading to electrical abnormalities (e.g., reentrant ventricular arrhythmias) and mechanical dysfunction (e.g., heart failure). The BZ, consisting of a mixture of viable myocytes and fibrotic scar, facilitates slow conduction or reentry and is believed to serve as the substrate for ventricular tachyarrhythmias (Ursell et al., 1985; de Bakker et al., 1988). Furthermore, premature ventricular contractions that can initiate ventricular fibrillation have been shown to elicit from the BZ (Marrouche et al., 2004). Consequently, catheter-based ablation of the BZ is one strategy used to manage ventricular arrhythmias (Marchlinski, 2008) and up to 68% of successful ablation sites reside in the BZ (Verma et al., 2005). Thus, accurate characterization of the BZ is important for developing microstructurally realistic models of cardiac electrophysiology that may aid in identifying of the location of the arrhythmogenic substrate prior to catheter ablation. Previous work by Ashikaga et al. has also suggested that the BZ is characterized by abnormal mechanics (Ashikaga et al., 2005). Therefore, in conjunction with the findings in this study, the BZ exhibits altered mechanics, electrophysiology, and microstructure.

The increase in BZ ADC likely results from the mixture of fibrotic scar and viable myocytes, which increases the extracellular volume and decreases the cellular volume. The larger increase in ADC within the infarct region corresponds to fibrotic scar fully replacing viable myocytes in this region and accords with previous studies observing higher rates of diffusion in infarct regions (Chen et al., 2003; Wu et al., 2007).

The observed decreases in FA within the BZ and infarct regions likely results from two remodeling phenomena: (1) the replacement of myocyte architecture with a more isotropic extracellular collagen network due to replacement fibrosis and; (2) an increase in myofiber disarray, which produces an apparent increase in the isotropy of water diffusion. Such observations of fiber disarray in infarcted myocardium have previously been observed (Chen et al., 2003; Strijkers et al., 2009). Decreased FA within the infarct from this study confirms observations of lower diffusion anisotropy within infarcted myocardium from previous studies (Chen et al., 2003; Wu et al., 2007). The observed decrease in the pairwise comparison of tissue mode within the BZ and infarct may also result from an increase in fiber disarray as local diffusion shifts away from linear isotropy toward orthotropic or planar diffusion. These changes in ADC, FA, and tissue mode could be used to refine computational models of the heart by proportionally adjusting tissue conductivity (lower in regions of higher ADC) and anisotropy (lower electromechanical anisotropy in regions of lower FA).

Differences in ADC and FA between remote myocardium from infarcted hearts and normal myocardium from control hearts were not statistically significant. Previous studies have shown wall thinning and reduced strain (Weisman et al., 1985, 1988; Bogaert et al., 2000), as well as myocyte lengthening and hypertrophy in remote myocardium when compared to normal controls (Anand et al., 1997). However, collagen volume fraction is not significantly different in remote and control myocardium (Marijianowski et al., 1997), which is consistent with the similar ADC and FA in those regions. The observed tissue mode decrease from normal to remote myocardium indicates an increase in sheet-like structure, which may facilitate the previously observed remote compensatory hyperfunction and increased wall thickening (Sutton and Sharpe, 2000).

Although previous studies have evaluated microstructural remodeling in post-infarct myocardium using DT-MRI (Chen et al., 2003; Wu et al., 2007, 2011; Strijkers et al., 2009), they have used imprecise methods to segment regions into infarct, BZ, and remote regions using short-axis T2-weighted (non-diffusion weighted) images from the DT-MRI experiment. Our study is the first to use LGE MRI to create accurate segmentations of infarct and BZ on a voxel by voxel basis. Previous studies of myocardial remodeling after infarction using DT-MRI have also used *t*-tests to compare DT parameters between regions, however, due to non-Gaussian data sets, spatially correlated data, and unequal variances between data sets standard methods are not appropriate. By de-correlating the data and using bootstrapping methods this is the first study to correctly quantify statistically significant microstructural remodeling of BZ and infarcted myocardium using DT-MRI. These imaging and statistical methods establish that the BZ and infarct are unique microstructural environments.

Methods to evaluate cardiac microstructure with *in vivo* DT-MRI continue to evolve (Aliotta et al., 2017; Nielles-Vallespin et al., 2017). Moving forward studies could be performed that compare the BZ as apparent on *in vivo* LGE MRI to *in vivo* DT-MRI microstructural remodeling. Such studies could lead to important changes to the methods being used to build patient-specific computational models of cardiac disease.

### Limitations

This study used a combination of LGE MRI and DT-MRI to quantify microstructural remodeling in the post-infarct porcine heart, but histological data is not available to confirm the microstructural remodeling results. However, the histological characterization of BZ infarcts has been previously performed in detail. The purpose of this study was to identify MRI-based measures of microstructural remodeling that may aid more accurate computational model construction. Futhermore, due to resolution constraints, voxels of intermediate SI designating BZ may arise from areas containing an interdigitated mixture of fibrosis and viable myocytes or from adjacent dense infarct and viable myocardium with a single well-defined border (Schelbert et al., 2010). The data from this study was not amenable to distinguishing between these two possible origins of intermediate SI and both were defined as BZ, but may have different electrophysiologic implications. Improvements in DT-MRI resolution and imaging methods may alleviate this ambiguity in BZ segmentation, however, the resolution achieved in this study is similar to those used in previous porcine DT-MRI studies (Wu et al., 2007, 2011).

## Conclusion

DT-MRI can identify regions of significant microstructural remodeling in the BZ that are distinct from both remote and infarcted myocardium.

## Author contributions

All authors contributed to the study conception and design. GK, MV, and DE were responsible for acquisition of data. All authors contributed to analysis and/or interpretation of data. drafting/revising the manuscript for intellectual content; and final manuscript approval.

### Conflict of interest statement

The authors declare that the research was conducted in the absence of any commercial or financial relationships that could be construed as a potential conflict of interest.

## Supplementary material

Supplementary Movie 1 Three-dimensional whole-infract visualization of diffusion tensor shape and orientation rendered with superquadric glyphs.
